# Renal Urotensin II System Plays Roles in the Regulation of Blood Pressure in Dahl Salt-Resistant Rat

**DOI:** 10.1155/2016/9146870

**Published:** 2016-12-19

**Authors:** Fei Wu, Guanjong Chen, Aihua Zhang, Yang Yu, Minhua Fan, Chaoshu Tang

**Affiliations:** ^1^Department of Nephrology, Peking University Third Hospital, Beijing, China; ^2^Department of Gynecology and Obstetrics, Peking University Third Hospital, Beijing, China; ^3^Department of Pathology and Physiology, Peking University Health Science Center, Beijing, China

## Abstract

*Introduction*. Dahl salt-resistant (SR) animal models are similar to peritoneal dialysis patients with fluid volumes overload with normal blood pressure in hemodynamic profiles. We will verify the roles of UII in the regulation of blood pressure in these animal models.* Methodology*. The Dahl salt-sensitive (SS) and SR rats and UII receptor gene knocked out (KO) mice were placed on a high-salt diet. Renal tissues were performed for the expression of UII in Dahl groups.* Results*. After high-salt diet for 6 weeks, the systolic blood pressure (SBP) in SR group was significantly lower, accompanied with higher urinary UII levels, higher 24-hour urinary sodium excretion, and higher urinary creatinine clearance in the SR rats in comparison to SS group. The expressions of UII and UT were both upregulated in the kidney tissues of SR group in comparison to SS group (*P* < 0.05). After high-salt diet for 8 weeks, the SBP of the KO group is significantly higher than that of the wild type group.* Conclusion*. We first demonstrate that renal UII system can play important roles in the regulation of blood pressure in Dahl SR rats which can be highly correlated to its effect on renal tubular sodium absorption.

## 1. Introduction

Urotensin II (UII) is a vasoactive polypeptide originally isolated from fish spinal cord and later in humans. It is the agonist for the orphan receptor GPR14 presently known as UII receptor (UT). UII is one of the most potent vasoconstrictors in existence [1]. In addition to its vasoconstrictive property, UII can also cause vasodilation effect in small resistant arteries [2]. In our previous study, we found that the concentration of plasma UII was significantly elevated in peritoneal dialysis patients when the systolic blood pressure was lower than 130 mmHg. The study also showed that UII concentration in normotensive patient with volume overload was increased dramatically in comparison to hypertensive patients with volume overload. Our results hinted that UII may play a role in vasodilatation and blood pressure regulation when there is an increase in volume [3].

However, our previous study was clinical in nature, and we were not able to account for all the possible confounding factors that could affect our findings. The underlying mechanism of blood pressure regulation was unclear. In order to validate the association between the increase in UII expression and its function in regulating blood pressure, we conducted this study using Dahl salt-resistant and salt-sensitive rats, selectively bred models developed by Dr. Dahl et al. for susceptibility or resistance to the hypertensive effect of high-salt diet. The renal vascular resistance of rats on a low-salt diet was less in Dahl salt-sensitive than Dahl salt-resistant rats [4]. After feeding high-salt diet (8% NaCl) for 4 weeks, Dahl salt-resistant rats had decreased renal vascular resistance. The slope of pressure-renal blood flow curves also increased compared to the pressure-renal blood flow curves of rats on low-salt diet (0.4% NaCl) [5]. The hemodynamic profiles of Dahl salt-resistant and Dahl salt-sensitive rats are very similar to those of volume-resistant and volume-sensitive dialysis patients. For further demonstration, model of UT gene knockout (KO) mice was established. By choosing these particular animal models, we were able to conduct a study that demonstrates the role of UII in the regulation of blood pressure.

## 2. Materials and Methods

### 2.1. Animal Experiments Protocol

Experiments were performed on 7-week-old male Dahl salt-sensitive rats (purchased from Vital River Laboratory Animal Technology Co., Ltd.) and Dahl salt-resistant rats (imported from Harlan Laboratory™, Laboratory Animal Medicine North America, USA), weighing between 200 and 250 grams. The rats were housed under standard condition. The experimental protocols were approved by the Biological Medical Ethics Committee of the Peking University Health Science Center (Approval number: LA2012-73). Experimental animals were divided into two groups (8 rats for each group): salt-sensitive (SS) group and salt-resistant (SR) group. All rats were fed on high-salt (8% sodium chloride) diet (Beijing HFK Bioscience Company) for 6 weeks. Blood and urine samples were obtained every two weeks and stored at −80°C for testing blood UII, urinary UII, urinary sodium concentration, and 24-hour sodium excretion. Rat glomerular filtration rate was estimated by creatinine clearance. Blood pressures of each rat were measured every week. At the end of the study, all rats were sacrificed under sodium pentobarbital anesthesia (50 mg/kg). Their tissues were harvested, and kidneys were either obtained for storage in liquid nitrogen for RNA and protein extraction or fixed in 10% neutral buffered formalin that was subsequently embedded into paraffin for immunohistochemical study.

*Interfered Study for Intravenous Injection of UII and UT Antagonist (Urantide) in Dahl Rat*. The UT antagonist (urantide) (10 mmol/kg) was injected by bolus intravenously at 0 minutes in SR rat and systolic blood pressure was measured at 5 minutes, 10 minutes, 15 minutes, 30 minutes, and 40 minutes after injection, and UII (1 nmol/kg) was injected by bolus intravenously at 0 minutes in SS rat and systolic blood pressure was measured at 5 minutes, 10 minutes, 15 minutes, 30 minutes, and 40 minutes after injection.

### 2.2. Measurement of Blood Pressure

Arterial blood pressures for all the conscious rats and mice were measured every week in the early morning. The blood pressures were measured by noninvasive tail-cuff method, and the measurements were repeated 10 times for each rat. Final blood pressure recorded for each rat is the mean of the 10 measurements.

### 2.3. Measurement of Plasma and Urinary UII

Whole blood and urine were collected in EDTA tubes and immediately transported to the analytical laboratory on ice. Blood serum was separated via 3000 rpm (1008 ×g) centrifugation for 10 min at 4°C. The supernatants were collected and stored at −80°C until the time of assay. The minimum sensitivity of UII radioimmunoassay was 0.1 pg/mL; the intra-assay and interassay coefficients of variation were 4.6% and 8.2%, respectively. Urinary UII level was corrected by urinary creatinine. Plasma and urinary UII were measured using a radioimmunoassay according to our previous publication and related literatures [3].

### 2.4. Immunochemistry Staining of Paraffin Embedded Kidney Tissue and Aortic and Mesenteric Small Arteries Tissue

The kidney tissues and aortic tissues were sectioned at a thickness of 10 *μ*m. For immunohistochemistry, 5% hydrogen peroxide was used to deplete endogenous peroxidase activity. Following preincubation with 5% bovine serum albumin for 30 min to prevent nonspecific staining, the sections were incubated with UII polyclonal antibody (Biosynthesis Biotechnology Company, bs-7521R, Beijing, China) and UT polyclonal antibody (Abcam, ab150584, Hong Kong) with dilutions of 1 : 300 and concentration of 15 *μ*g/mL, respectively, at 4°C overnight. The sections were then incubated with horseradish peroxidase-coupled goat anti-rabbit IgG antibody for 20 min, followed by incubation with strep-avidin-biotin-peroxidase complex (SABC) for 20 min at 37°C. The peroxidase was visualized by incubation with 3,3′-diaminobenzidine (DAB) in the dark for 3 min. The sections were counterstained with hematoxylin, dehydrated, and observed under a light microscope. Negative controls were established using PBS as a substitute for first antibody. Positive staining was indicated by brown deposits. For semiquantitative analysis, 10 high-power microscope fields were randomly selected, and the pathological image analysis system was used to calculate the integral optical density (IOD) of positive staining for UII and UT.

### 2.5. RNA Isolation and Quantitative Real-Time PCR Analysis

Total RNA was extracted from the kidneys of both baseline and high-salt fed salt-sensitive and salt-resistant rats using TRIzol Reagent (Invitrogen Ltd.) and reverse-transcribed into first-strand complementary DNA from 1 *μ*g of DNase-treated RNA using GoScript TM Reverse Transcription System (A5001, Promega Co., USA). A real-time quantitative PCR method was used to detect the changes in UII and UT mRNA levels. Primers for rat UII, UT, and housekeeping genes *β*-acting were designed using Primer Express software (Applied Biosystems). Quantitative real-time PCR was carried out in a total volume of 20 *μ*L using a Bio-Rad iQ5 detector (Applied Biosystems) to determine the threshold cycle (Ct) value. Each sample was run and analyzed in triplicate. For the UII gene, the forward primers were 5′-GAGCAGACACCCAGCCAG-3′ and the reverse primer was 5′-CTCCTCCAGAGCCCGAAG-3′. For the UT gene, the forward primers were 5′-CCATAATGAGCAGCGAAC-3′; the reverse primer was 5′-CCCAGAAGAGAAGGACGA-3′. For *β*-actin control, the forward primer was 5′-GGTCCACACCCGCCACCAGTT-3′; the reverse primer was 5′-ACCCATACCCACCATCACACCCTG-3′. Relative quantitation values were calculated using fold changes in the target gene related to the expression of *β*-actin. Three-step real-time PCR of denaturing, annealing, and extension reactions proceeded for 40 cycles at 15 s at 95°C, 1 min at 53°C [UII, UT], and 30 s at 72°C. For *β*-actin, the annealing temperature is 60°C.

### 2.6. Western Blot Analysis

Proteins were extracted from the kidneys of SS group and SR group. Western blot analysis of UT was performed. Western blot analysis for UII was not performed because there is no anti-rat UII antibody. The protein samples were denatured at 95°C for 5 min and separated on a 10% SDS-PAGE gel before transferring them to NC membranes (Applygen Technologies Inc., Beijing, China). Membranes were subsequently incubated with primary rabbit polyclonal anti-GPCR GPR14 antibodies (1 : 1000; ab156003, Abcam) and anti-*β*-actin antibodies (1 : 500 TA-09, Zhongshan Gold Bridge Biotechnology Co., Ltd., Beijing, China) overnight at 4°C, followed by incubation with horseradish peroxidase- (HRP-) conjugated anti-rabbit antibodies (1 : 500, Zhongshan Gold Bridge Biotechnology Co., Ltd., Beijing, China). Semiquantitative grayscale intensity was generated with Odyssey software v1.

### 2.7. UT Knockout Mice PCR Design

The UT gene knockout (KO) mouse strain used for this research project was created from ES cell clone 12922A-B6, generated by Regeneron Pharmaceuticals, Inc., and made into live mice by the KOMP Repository (https://www.komp.org/) and the Mouse Biology Program (https://www.mousebiology.org/) at the University of California, Davis. Then the four heterozygous male mice were imported to Peking University Third Hospital and matched and rotated with young female C57Bl/6 mice and bred eight generations; PCR male heterozygous mice were crossed with C57B1/6 females to give N1F0 offspring, which were subsequently intercrossed to generate N1F1 offspring. In addition, NEFF offspring were successively backcrossed to C57B1/6 females to generate N5F0 mice. These were intercrossed to create an N5F1 population. UT gene knockout mice were identified by PCR methods. Kidneys of wild type and UT gene KO mice were cut into small pieces and placed into polypropylene tubes.

*Genotype Identification*. The following is genotype identification according to the Mouse Biology Program, University of California, Davis,

Primers of Reg-NeoF: 5′-GCAGCCTCTGTTCCACATACACTTCA-3′
Reg-Uts2r-R: 5′-CTCTCAGATCTCTCAGCTACCTGCC-3′
Reg-Uts2r-wtR: 5′-CTTGAAGGAAGCTTGCTGGGATAGC-3′
Reg-Uts2r-wtF: 5′-ATTGGGCTGCTCTATATCCGTCTGG-3′
Genotype forward primer reverse primer amplicon size (bp): knockout, Reg-NeoF Reg-Uts2r-R, 756 bp; wild type, Reg-Uts2r-wtF Reg-Uts2r-wtR, 63 bp

Toes of newborn mice within 6 weeks were cut for DNA extraction (DNA extraction kit, D3396-01 OMEGA).

Amplification 756 bp means urotensin II receptor is knocked out and 63 bp means wild type; both 756 bp and 63 bp mean heterozygotes of UT gene knockout KO mouse as well as mRNA and protein expression of UT KO group, according to our previous publications [6].

### 2.8. Phenotypic Analysis

Blood glucose and blood pressure are tested using method described above. Blood samples are obtained to perform blood routine examination.

### 2.9. High-Salt Induced Blood Pressure Increase in KO Mouse

6 KO mice and 6 wild type (WT) mice aging 6 weeks and weighing between 21 and 23 grams were selected and were housed under standard SPF condition. All mice were fed on high-salt (8% sodium chloride) diet (Beijing HFK Bioscience Company) without limit on water intake for 8 weeks. Blood and urine samples were obtained every two weeks and stored at −80°C for testing blood UII, urinary UII, urinary sodium concentration, and 24-hour sodium excretion. The volume of urine and duration of attaining the urine were recorded for mice GFR calculation. Mouse glomerular filtration rate was estimated by creatinine clearance (creatinine clearance (mL/min) = urinary creatinine concentration/blood creatinine concentration *∗* 24-hour urine volume/1440). Blood pressures of each mouse were measured every week regularly.

### 2.10. Statistical Analysis

Statistical analysis was performed using SPSS software, version 13.0 (SPSS Inc., Chicago, IL, USA). Continuous variables are expressed as mean ± SD. Independent-samples *t*-test or one-way ANOVA test was applied in statistical analysis. LSD was performed for post hoc analysis after we use ANOVA. *P* values below 0.05 were accepted to be significant.

## 3. Results

### 3.1. Changes of Systolic Blood Pressure, Plasma UII, and Urinary UII between SR Rats and SS Rats

**Figure 1:**
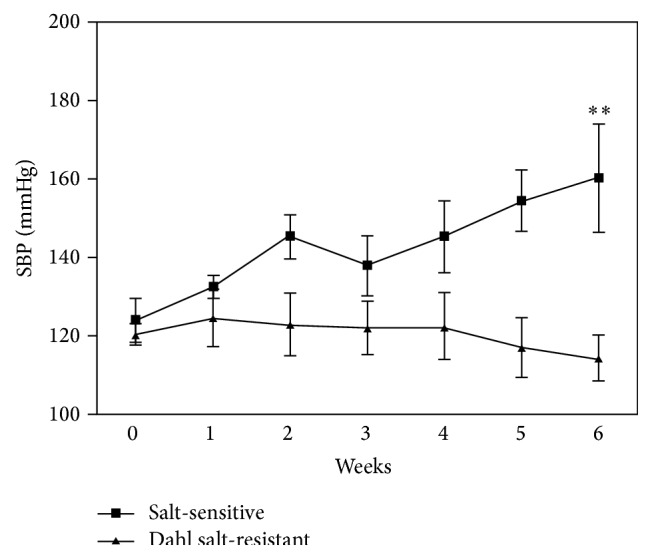
Figure 1 showed changes of systolic blood pressure between SR rats and SS rats. ^*∗∗*^ *P* < 0.01 versus SR rats.

At baseline, systolic blood pressure in SR group was 120 ± 3 mmHg and systolic blood pressure in SS group was 124 ± 6 mmHg. There was no significant difference in the systolic blood pressures between the two groups at baseline During the 6 weeks of high-salt forage feeding; the systolic blood pressure in SS group increased gradually, whereas the systolic blood pressure in SR group was stable. At the end of 6 weeks, the systolic blood pressure in SS group was significantly higher compared to SR group (160 ± 13 mmHg versus 114 ± 6 mmHg, *P* < 0.01) (Figure 1).

There was no difference in the baseline weight between SR group and SS group (213 ± 6.8 g versus 208 ± 7.7 g, *P* > 0.05), while there was also no difference in the weight between SR group and SS group (308 ± 30 g versus 309 ± 18 g, *P* > 0.05) after six weeks of high-salt diet.

**Figure 2:**
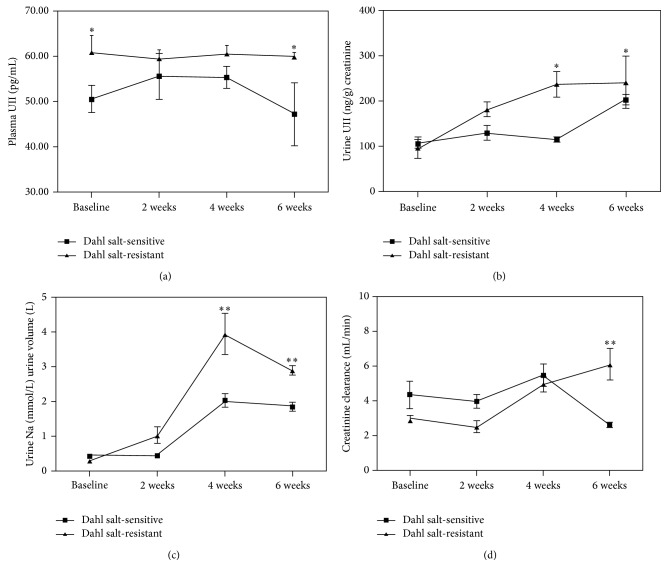
Figure 2 demonstrated the changes of plasma UII, urinary UII, 24-hour urinary sodium excretion, and creatinine clearance between SR rats and SS rats after high-salt forage feeding for 6 weeks. ^*∗*^ *P* < 0.05 versus SS group; ^*∗∗*^ *P* < 0.01 versus SS group (a, b, c, and d).

At baseline, plasma UII level was significantly higher in SR group than in SS group (61 ± 5.9 pg/mL versus 51 ± 2.4 pg/mL, *P* = 0.015). Plasma level of UII continued to be significantly higher without continuous increase in SR group in comparison to SS group after high-salt forage feeding for 6 weeks (60 ± 6.4 pg/mL versus 52 ± 12.4 pg/mL, *P* < 0.03) (Figure 2(a)).

There was no difference in urinary UII (corrected by urinary creatinine) between SR group and SS group at baseline. However, the urinary level of UII was significantly increased in the SR group in comparison to SS group after they were fed high-salt diet for 4 weeks (181.3 ± 83.9 ng/g versus 136.7 ± 45.4 ng/g, *P* < 0.05) (Figure 2(b)).

There was no difference in 24-hour urinary sodium excretion and creatinine clearance between SR group and SS group at baseline (0.3 ± 0.1 mmol versus 0.5 ± 0.2 mmol, *P* > 0.05; 3.5 ± 0.2 mL/min versus 4.2 ± 0.7 mL/min, *P* > 0.05). However, the 24-hour urinary sodium excretion was significantly increased in the SR group compared with SS group after high-salt diet for 4 weeks (3.8 ± 0.6 mmol versus 1.9 ± 0.2 mmol, *P* < 0.01) and 6 weeks (3.1 ± 0.2 mmol versus 1.7 ± 0.2 mmol, *P* < 0.01), and 24-hour creatinine clearance was significantly increased in the SR group compared with SS group after high-salt diet for 6 weeks (6.1 ± 0.8 mL/min versus 2.8 ± 0.2 mL/min, *P* < 0.01) (Figures 2(c) and 2(d)).

### 3.2. Analysis of Immunohistochemistry for UII and UT Expressions in Kidney Tissue, Aortic Tissue, and Mesenteric Small Arteries Tissue

**Figure 3:**
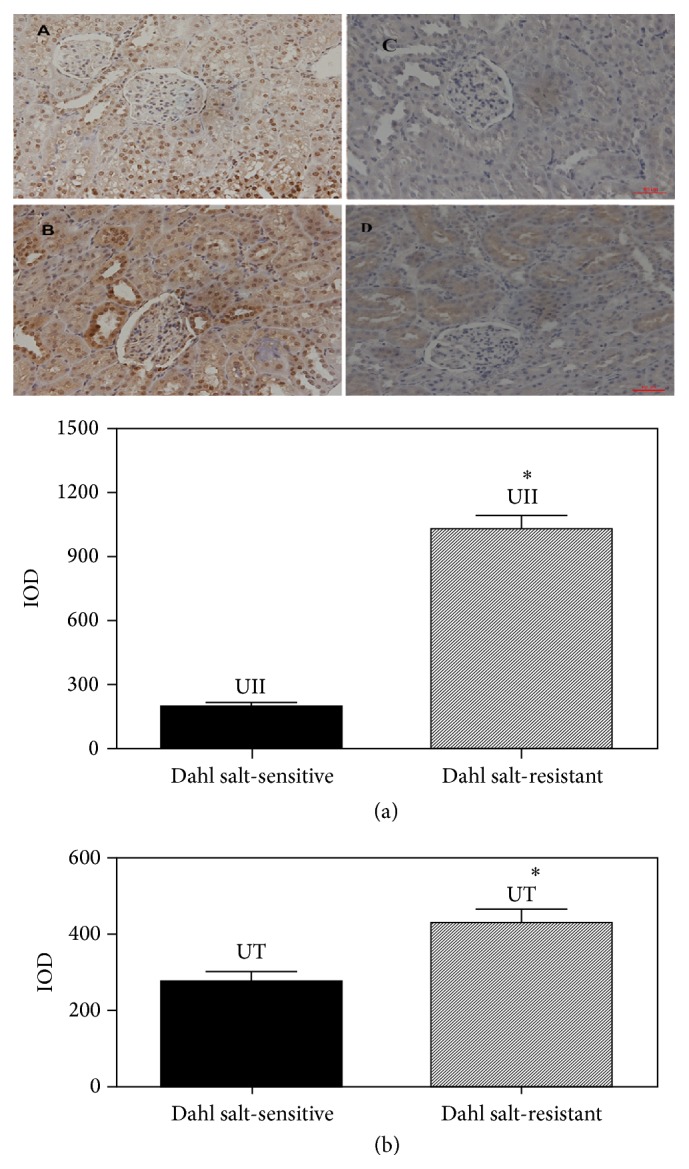
Figure 3 showed analysis of immunohistochemistry for UII (A and B) and UT expressions (C and D) in kidney tissue in SS and SR rats. The expressions of UII and UT were present predominantly in renal tubular epithelium and less in glomerulus (A, B, C, and D). ^*∗*^ *P* < 0.01 versus IOD of SS rats (a and b).

Analysis of immunohistochemistry in kidney tissue showed that expressions of UII and UT were present predominantly in renal tubular epithelium and less in glomerulus (Figures 3(A), 3(B), 3(C), and 3(D)). Expressions of UII and UT were more intense in the SR group compared to the SS group after 6 weeks of high-salt diet (Figures 3(a) and 3(b)). However, there was no difference in expressions of UII and UT in aortic tissues between SR group and SS group (data not shown), while there was significantly higher expression of UT in intima of mesenteric small arteries in SR rats in comparison to SS rats (data not shown).

### 3.3. Comparison of mRNA Expression of UII and UT in Kidney between SR Group and SS Group by Real-Time PCR

**Figure 4:**
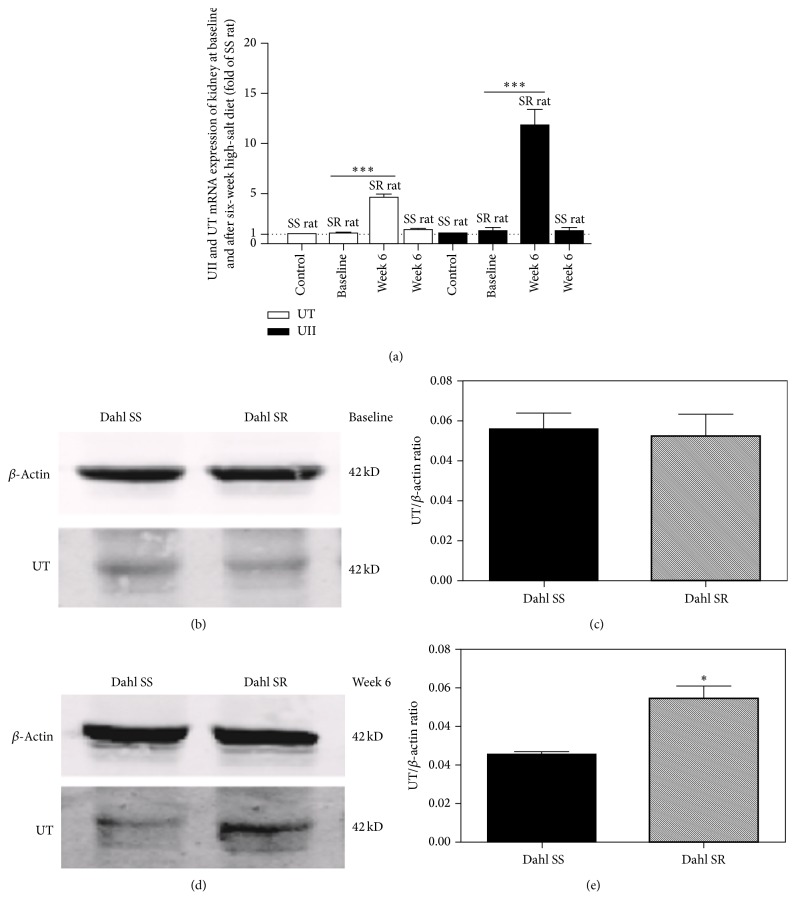
Figure 4 showed comparison of mRNA expression (a) and protein of UII and UT in kidney (b, c, d, and e) by real-time PCR and Western blot between SR group and SS group at baseline and after six weeks of high-salt diet. ^*∗∗∗*^ *P* < 0.001 versus SS group (versus SS rats, week 6, in (a)). *∗* indicates *P* < 0.05 versus SS rats.

At baseline, there was no difference in mRNA expressions of UII and UT between SR group and SS group. However, UII and UT mRNA expressions of Dahl SR group were significantly increased compared to Dahl SS group after 6 weeks of high-salt feeding. UII expression in the kidney of Dahl SR group was 11.97-fold higher than the Dahl SS group (*P* < 0.001). As for UT expression, the SR group is 4.63-fold higher compared to the SS group (*P* < 0.001) (Figure 4(a)).

*Comparison of Protein Expressions of UII in the Kidneys between SR Group and SS Group by Western Blot*. Western blot analysis showed that the expression (normalized by *β*-actin) of UT had no difference between two groups [(SR) 0.060 ± 0.006 versus (SS) 0.058 ± 0.008, *P* > 0.05] at baseline (Figures 4(b) and 4(c)). After high-salt feeding for 6 weeks, the protein expression of UT of SR group was significantly increased when compared to that of the SS group (0.059 ± 0.008 versus 0.036 ± 0.001, *P* < 0.05) (Figures 4(d) and 4(e)).

### 3.4. Changes of Systolic Blood Pressure in Dahl Rats after UII and UT Antagonist Injected Intravenously

**Figure 5:**
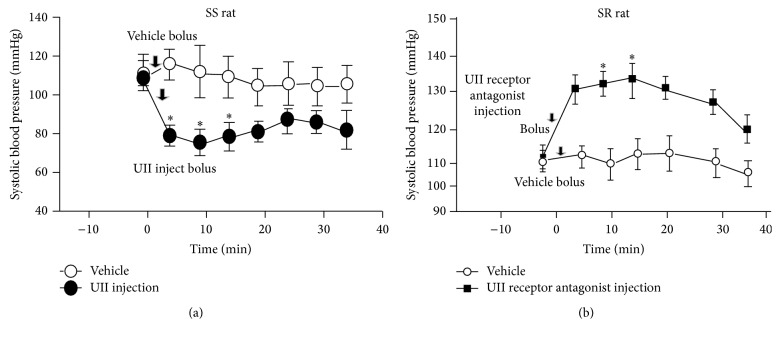
Figure 5 showed changes of systolic blood pressure in Dahl rats after UII and antagonist injected intravenously by bolus. UII (1 nmol/kg) was injected intravenously by bolus in SS rats (a), while the antagonist (urantide) (10 mmol/kg) was injected intravenously by bolus in SR rats (b). ^*∗*^ *P* < 0.01 versus vehicle.

UII (1 nmol/kg) was injected intravenously by bolus at 0 minutes in SS rat and systolic blood pressure was decreased significantly at 5 minutes, 10 minutes, and 15 minutes after injection (Figure 5(a)), while the UT antagonist (urantide) (10 mmol/kg) was injected intravenously by bolus at 0 minutes in SR rat and systolic blood pressure was significantly increased at 10 minutes and 15 minutes after injection (Figure 5(b)).

### 3.5. Genotype Identification of UT Knockout Mice

**Figure 6:**
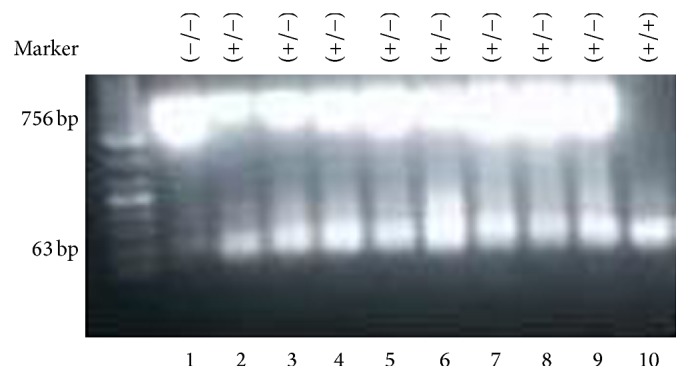
Lane 1 (−/−) is homozygotes of UT gene knockout (KO group) genotype mouse; lanes 2–9 (+/−) are heterozygotes of UT gene knockout mice; lane 10 (+/+) is wild type (WT) mouse.

Genotype identification of UT gene knockout mice was performed by PCR according to our previous publications [6]. Lane 1 in Figure 6 (−/−) represents homozygotes of UT gene knockout (KO group) genotype; lanes 2 and 9 (+/−) represent heterozygotes of UT gene knockout; lane 10 (+/+) represents wild type (WT).

### 3.6. Phenotypic Analysis

Blood glucose of both types is within physiological range and there is no significant difference between wild type and KO mice (6.9 ± 0.77 mmol/L versus 7.7 ± 0.22 mmol/L, *P* > 0.05).

There was no significant difference in systolic blood pressure (SBP) (102 ± 7 versus 103 ± 11 mmHg, *P* > 0.05), diastolic blood pressure (DBP) (60 ± 9 mmHg versus 63 ± 5 mmHg, *P* > 0.05), mean blood pressure (MBP) (74 ± 9 mmHg versus 76 ± 7 mmHg, *P* > 0.05), and the heart rate (499 ± 22 times/min versus 529 ± 95 times/min, *P* > 0.05) between WT and KO groups (Figure 6) at baseline. Glucose and blood routine examination showed no significant difference at baseline.

### 3.7. Comparison of Blood Pressure and 24-Hour Urinary Sodium Excretion and Creatinine Clearance after High-Salt Diet

**Figure 7:**
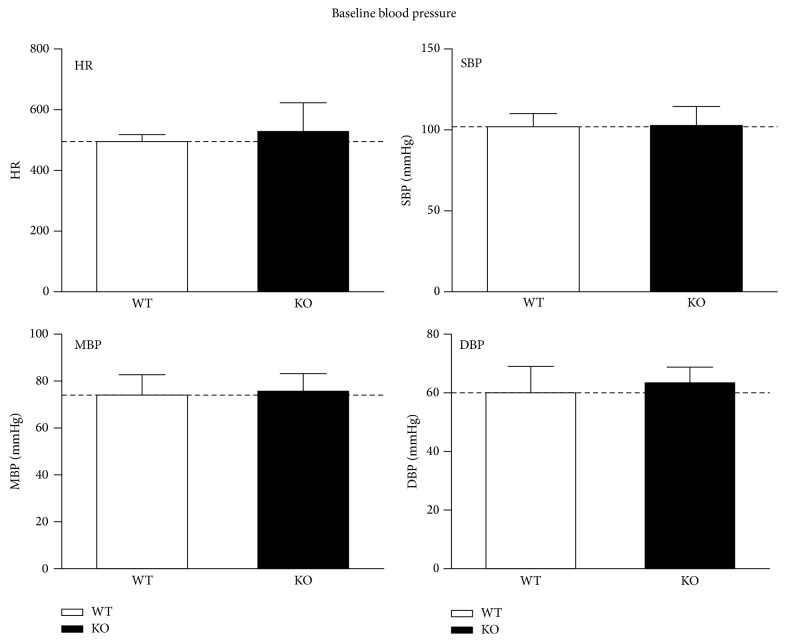
Figure 7 showed phenotypic analysis of KO mice; there was no significant difference in systolic blood pressure (SBP), diastolic blood pressure (DBP), mean blood pressure (MBP), and the heart rate at baseline. Glucose and blood routine examination showed no significant difference at baseline.

SBP in KO mice was significantly elevated since the 4th week after high-salt feeding in comparison to wild type mice (113 ± 3 mmHg versus 102 ± 4 mmHg, *P* = 0.003) and so was it at 5th week (108 ± 6 mmHg versus 97 ± 4 mmHg, *P* = 0.021), at the 6th week (104 ± 3 mmHg versus 96 ± 3 mmHg, *P* = 0.003), at the 7th week (107 ± 5 mmHg versus 97 ± 5 mmHg, *P* = 0.049), and at the 8th week (111 ± 1 mmHg versus 98 ± 7 mmHg, *P* = 0.015) (Figure 7).

**Figure 8:**
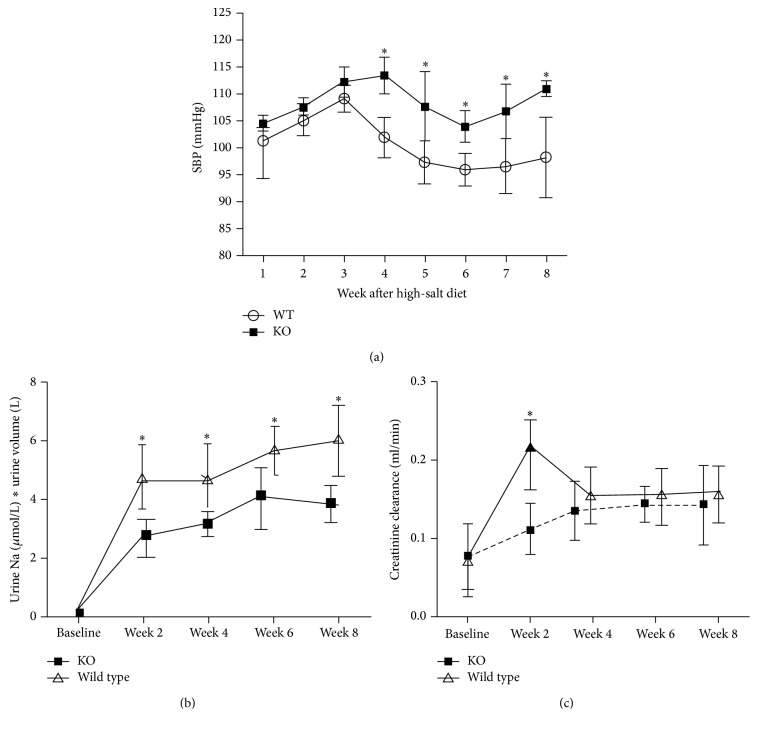
Figure 8 showed comparison of blood pressure (a) and 24-hour urinary sodium excretion (b) and creatinine clearance (c) after high-salt diet between KO mice and WT mice. ^*∗*^ *P* < 0.05 versus WT mice.

There was no difference in 24-hour urinary sodium excretion and creatinine clearance between WT group and KO group at baseline (0.2 ± 0.1 *μ*mol versus 0.2 ± 0.1 *μ*mol, *P* > 0.05; 0.07 ± 0.04 mL/min versus 0.07 ± 0.04 mL/min, *P* > 0.05). However, after high-salt diet at 2nd week and 4th week and at 6th week and 8th week, the 24-hour urinary sodium excretion was significantly decreased in the KO group compared with WT group at 2nd week (2.97 ± 0.4 *μ*mol versus 4.85 ± 0.5 *μ*mol, *P* < 0.03), at 4th week (3.11 ± 0.6 *μ*mol versus 4.29 ± 0.68 *μ*mol, *P* < 0.05), at 6th week (4.45 ± 0.57 *μ*mol versus 5.56 ± 0.58 *μ*mol, *P* < 0.05), and at 8th week (3.31 ± 0.42 *μ*mol versus 5.66 ± 0.66 *μ*mol, *P* < 0.01). 24-hour creatinine clearance was significantly decreased in the KO group compared with WT group after high-salt diet at 2nd week (0.23 ± 0.02 mL/min versus 0.11 ± 0.01 mL/min, *P* < 0.02); however, there was no significant difference in creatinine clearance at 4th week, 6th week, and 8th week (Figures 8(b) and 8(c)).

## 4. Discussion

UII is an important molecule in the pathophysiology of human diseases. Many studies showed that elevated plasma level of UII and increased expression of UII and UT can be identified in the tissues of numerous diseased conditions, including hypertension, preeclampsia, atherosclerosis, heart failure, pulmonary hypertension, diabetes, renal failure, and various metabolic syndromes [7, 8]. It is reported that UII is upregulated in the spontaneously hypertensive rat [9], which means that UII is mainly a vasoconstrictor [9, 10]; however, recent studies also showed that UII has vasodilatory effect. An example demonstrating the dilatory function of UII is that patients on hemodialysis with lower blood pressure were found with elevated UII level [11]. Studies of intravenous infusions of UII in animals also showed that UII can cause vasodilation [12].

In our previous study, we found that plasma UII concentration in normotensive patient with volume overload was increased dramatically in comparison to hypertensive patients with volume overload. This also indicates that UII has a vasodilatory effect. In order to verify the roles of UII in normotensive patients despite volume overload and its role in the regulation of blood pressure at the molecular level, we chose Dahl salt-resistant and salt-sensitive animal model to conduct further study. Our results showed that plasma UII level was markedly increased at baseline in Dahl SR rats compared with Dahl SS rats. After 6 weeks of high-salt diet, the plasma UII level was found to be continually elevated in Dahl SR rats in comparison to that of Dahl SS rats. Throughout this period, Dahl SR rats also remained normotensive, while Dahl SS developed hypertension. Meanwhile, systolic blood pressure in Dahl SR rats increased significantly after intravenous injection of urantide, while systolic blood pressure in Dahl SS rats decreased significantly after intravenous injection of UII. On the other hand, circulation of UII maintained high level and did not increase continuously in comparison to baseline; moreover, our results showed that the mRNA concentrations of UII and UT were increased in the kidney of SR rats after six weeks of high-salt diet. Additionally, the expressions of UII and UT (GPR14) were mainly located in the renal tubular epithelium of the SR group. We also found that urinary UII increased in SR group. Urinary UII mainly originates from the renal source [12], which is predominantly from the renal tubular epithelial cell. The elevation of urinary UII matches our finding that UII and its receptor are mainly found in the renal tubular epithelium. We found that plasma UII levels in SR rats were more stable compared to SS rats, while urinary UII levels were similar at baseline and increased in SR rats after salt loading. This is a very important phenomenon. Urinary UII comes from renal tubular epithelial cell (the evidence is that urotensin II has short half-life, and urinary UII level is significantly higher than plasma UII), while plasma UII mainly comes from vascular (small artery) endothelial cell (the evidence is previous reported higher UII expression in kidney, heart, etc., but plasma UII concentration is very low, pg/mL) [1, 2, 7], which means kidney UII system plays important roles in regulation of blood pressure in Dahl rats after salt loading.

Another example showing that UII and its receptor can have different functions among different types of tissues is that the UT presenting in smooth muscle can activate PKC which increases cytosolic Ca2+ level which leads to contraction [13]. As a part of the endothelium of peripheral vessels, UII and its receptor can cause vasodilatation through mediation by nitric oxide [13]. In our current study, we, however, did not find a difference in the expression of UII and UT in the aortic artery between the SR group and SS group. This may mean that there was no difference in the vasoconstrictive effect of circulation UII on aortic artery between the two groups (our in vitro study has verified this, data not shown). That gives a hint that renal UII may play more important roles in Dahl SR rats in the long term. It is well known that urotensin-related peptide (URP) is a paralog of UII in that it contains the six amino acid ring structures found in UII [14]. Although UII and urotensin-related peptide are implicated as bioactive factors capable of modulating cardiovascular status, we still need to investigate expressions and roles of UII related peptide in SR and SS model in our further study.

Whereas systolic blood pressure in Dahl SR rats increased significantly after intravenous injection of urantide (UT antagonist), systolic blood pressure in Dahl SS rats decreased significantly within 15 min of intravenous injection of UII in our current study, which means that circulation of UII plays roles in Dahl rats in the short term.

In our UT knocked out animal model, when mice were fed the same high-salt forage, the blood pressure of KO mice is markedly elevated, while the wild type mice keep baseline blood pressure; this also verified that effect of UII on blood pressure. To the best of our knowledge, our results are the first to demonstrate that UII could play a crucial role in the regulation of blood pressure as demonstrated in Dahl SR rats.

It is important to note that the dual effects of UII in vasodilation and vasoconstriction can have different presentations across the various regions of the vascular beds and types of species. A study demonstrating this phenomenon showed that UII caused profound vasoconstriction in cynomolgus monkey but vasodilation in rat [15, 16]. Additionally, UII can induce significant vasodilatation in peripheral arteries, whereas it can induce vasoconstriction in aortic artery [13, 17]. This again showed that UII can serve different functions when presented in different types of setting. Its dual capabilities of vasoconstriction and vasodilation may serve as a critical factor in the regulation of blood pressure [18].

As a part of the endothelium of peripheral vessels, UII and its receptor can cause vasodilatation through mediation by nitric oxide [19]. In addition to its different effects across the various tissues, different concentrations of UII can have different effects on vessels. Infusion of low-dose UII can induce vasodilation but infusion of high-dose UII can induce vasoconstriction. Previous studies also showed that the same dosage of UII can have varied blood pressure-lowering effect under different settings. A dosage of 3–10 ng/kg UII can lower blood pressure by 10–20% in WKY and Sprague-Dawley rats, but the same UII dose can reduce blood pressure in Lewis rats by 50% [20]. Our current study demonstrates that UII (1 nmol/kg) can lower blood pressure in SS rats.

How does UII regulate blood pressure of Dahl SR rats on high-salt diet? Besides its constricted or dilated function on vessels, it is reported that UII has been shown to regulate epithelial sodium transport. In secretory tissues, such as opercular skin, UII inhibited active sodium and chloride transport [9]. These observations demonstrate that UII can directly affect epithelial ion transport in fish and influence body fluid homeostasis [21]. Zhang et al. [22] also reported that low-dose UII infusion can increase GFR and urinary sodium excretion in rat. In our current study, there was no difference in urinary sodium excretion between SR group and SS group at baseline. However, the urinary sodium excretion and creatinine clearance (glomerular filtration rate highly correlated with creatinine clearance) were significantly increased in the SR group in comparison to SS group after 6 weeks of high-salt diet. This indicates that the elevated UII renal expression can have a crucial role in the regulation of blood pressure as our study showed that high-salt diet can cause Dahl SR rats to have increased creatinine clearance, consequently increasing urinary sodium excretion. The creatinine clearance and urinary sodium excretion in mice with UT gene knockout were significantly decreased in comparison to wild type mice after high-salt diet. On the other hand, we also found that there was no significant increase in creatinine clearance in SR rats since 6th week; there already has been an increase in urinary sodium excretion, which means UII may not simply be attributed to increase in creatinine and indirect increase in urinary sodium excretion and it means UII has the direct inhibition of tubular ion transport independent of creatinine clearance. Urinary UII in SS rats is increased at 6 weeks after salt loading, but we found it did not reach significant difference compared with baseline UII levels in SS rats. We believe urinary UII increase in SS rats at 6 weeks after high-salt loading is to compensate for increasing salt excretion but SR rat sodium excretion increase more than SS rat (you can see our Figure 2(c)). And this is also verified by UT gene knocked out mice.

Under clinical setting, there is also strong evidence that UII has a role in regulating blood pressure. From our previous study, we showed that the concentration of UII in normotensive peritoneal dialysis patients with high volume (volume-resistant) was significantly higher than that of hypertensive peritoneal dialysis patients with high volume (volume-sensitive). Since the majority of peritoneal dialysis patients had no residual renal function, UII would not be able to regulate blood pressure through an effect on renal sodium excretion. This gave a hint that the main action of UII in these patients might be dilating the small arteries. This is consistent with our current results where there was significantly higher expression of UT in intima of mesenteric small arteries in SR rats in comparison to SS rats. Our past findings that peritoneal dialysis patients with normal blood pressure and volume overload had lower left ventricular mass index when compared to that of the patients with hypertension and volume overload [23] UII might also serve as a protective factor for the cardiovascular system. Based on the findings in our current study, our results also indicated that UII is a molecule with multiple purposes and functions. Renal UII plays more important roles in regulation of blood pressure in Dahl SR rats through increasing creatinine clearance and increasing urinary sodium excretion; however, circulation of UII may also play some roles in Dahl SR rats through dilating small arteries. In kidney and blood circulation, UII can dilate small artery and increase urinary sodium excretion. Concurrently, it can also serve as a protective factor for the cardiovascular system.

The mechanism involved in the upregulation of UII system in SR rats after salt loading which was defective in SS rats was unclear. They may have species variation and characteristic variation between SR and SS rats; on the other hand, the upstream signal for UII upregulation of kidney in SR rats after high-salt loading is not elucidated; it is worthy of investigation in the future.

## 5. Conclusions

The present results first demonstrate that renal UII can play more important roles in the regulation of blood pressure in Dahl SR rats through increasing creatinine and urinary sodium excretion by animal experiments, UT antagonist in Dahl SR rats, and UT gene knockout mice animal models. Besides this, circulation of UII also plays some part roles in regulation of blood pressure through dilating small artery.

## References

[1] Ames R. S., Sarau H. M., Chambers J. K. (1999). Human urotensin-II is a potent vasoconstrictor and agonist for the orphan receptor GPR14. *Nature*.

[2] Stirrat A., Gallagher M., Douglas S. A. (2001). Potent vasodilator responses to human urotensin-II in human pulmonary and abdominal resistance arteries. *American Journal of Physiology—Heart and Circulatory Physiology*.

[3] Bai Q., Zhang J., Zhang A.-H. (2012). Roles of human urotensin II in volume resistance hypertension in peritoneal dialysis patients. *Renal Failure*.

[4] Dahl L. K., Heine M., Tassinari L. (1962). Effects of chronic excess salt ingestion. Evidence that genetic factors play an important role in susceptibility to experimental hypertension. *The Journal of Experimental Medicine*.

[5] Fink G. D., Takeshita A., Mark A. L., Brody M. J. (1980). Determinants of renal vascular resistance in the Dahl strain of genetically hypertensive rat. *Hypertension*.

[6] Pang X., Bai Q., Wu F., Chen G., Zhang A., Tang C. (2016). Urotensin II Induces ER Stress and EMT and Increase Extracellular Matrix Production in Renal Tubular Epithelial Cell in Early Diabetic Mice. *Kidney and Blood Pressure Research*.

[7] Ross B., McKendy K., Giaid A. (2010). Role of urotensin II in health and disease. *American Journal of Physiology—Regulatory Integrative and Comparative Physiology*.

[8] Gibson A. (1987). Complex effects of Gillichthys urotensin II on rat aortic strips. *British Journal of Pharmacology*.

[9] Forty E. J., Ashton N. (2012). Ontogeny of the renal urotensin II system in the rat. *Experimental Physiology*.

[10] Douglas S. A., Ohlstein E. H. (2000). Human urotensin-II, the most potent mammalian vasoconstrictor identified to date, as a therapeutic target for the management of cardiovascular disease. *Trends in Cardiovascular Medicine*.

[11] Mosenkis A., Kallem R. R., Danoff T. M., Aiyar N., Bazeley J., Townsend R. R. (2011). Renal impairment, hypertension and plasma urotensin II. *Nephrology Dialysis Transplantation*.

[12] Gardiner S. M., March J. E., Kemp P. A., Davenport A. P., Bennett T. (2001). Depressor and regionally-selective vasodilator effects of human and rat urotensin II in conscious rats. *British Journal of Pharmacology*.

[13] Tasaki K., Hori M., Ozaki H., Karaki H., Wakabayashi I. (2004). Mechanism of human urotensin II-induced contraction in rat aorta. *Journal of Pharmacological Sciences*.

[14] Mori M., Fujino M. (2004). Urotensin II-related peptide, the endogenous ligand for the urotensin II receptor in the rat brain. *Peptides*.

[15] Katano Y., Ishihata A., Aita T., Ogaki T., Horie T. (2000). Vasodilator effect of urotensin II, one of the most potent vasoconstricting factors, on rat coronary arteries. *European Journal of Pharmacology*.

[16] Douglas S. A., Sulpizio A. C., Piercy V. (2000). Differential vasoconstrictor activity of human urotensin-II in vascular tissue isolated from the rat, mouse, dog, pig, marmoset and cynomolgus monkey. *British Journal of Pharmacology*.

[17] Bottrill F. E., Douglas S. A., Hiley C. R., White R. (2000). Human urotensin-II is an endothelium-dependent vasodilator in rat small arteries. *British Journal of Pharmacology*.

[18] Zhu Y.-C., Zhu Y.-Z., Moore P. K. (2006). The role of urotensin II in cardiovascular and renal physiology and diseases. *British Journal of Pharmacology*.

[19] Gray G. A., Jones M. R., Sharif I. (2001). Human urotensin II increases coronary perfusion pressure in the isolated rat heart: potentiation by nitric oxide synthase and cyclooxygenase inhibition. *Life Sciences*.

[20] Disa J., Floyd L. E., Edwards R. M., Douglas S. A., Aiyar N. V. (2006). Identification and characterization of binding sites for human urotensin-II in Sprague-Dawley rat renal medulla using quantitative receptor autoradiography. *Peptides*.

[21] Abdel-Razik A. E., Forty E. J., Balment R. J., Ashton N. (2008). Renal hemodynamics and tubular actions of urotensin II in the rat. *Journal of Endocrinology*.

[22] Zhang A. Y., Chen Y.-F., Zhang D. X. (2003). Urotensin II is a nitric oxide-dependent vasodilator and natriuretic peptide in the rat kidney. *American Journal of Physiology—Renal Physiology*.

[23] Cheng L.-T., Tian J.-P., Tang L.-J. (2008). Why is there significant overlap in volume status between hypertensive and normotensive patients on dialysis?. *American Journal of Nephrology*.

